# Sensing Phosphatidylserine in Cellular Membranes

**DOI:** 10.3390/s110201744

**Published:** 2011-01-28

**Authors:** Jason G. Kay, Sergio Grinstein

**Affiliations:** Program in Cell Biology, Hospital for Sick Children, 555 University Ave., Toronto, ON M5G1X8, Canada; E-Mail: Jason.Kay@sickkids.ca

**Keywords:** phosphatidylserine, C2 domain, GFP, membrane dynamics, fluorescence microscopy

## Abstract

Phosphatidylserine, a phospholipid with a negatively charged head-group, is an important constituent of eukaryotic cellular membranes. On the plasma membrane, rather than being evenly distributed, phosphatidylserine is found preferentially in the inner leaflet. Disruption of this asymmetry, leading to the appearance of phosphatidylserine on the surface of the cell, is known to play a central role in both apoptosis and blood clotting. Despite its importance, comparatively little is known about phosphatidylserine in cells: its precise subcellular localization, transmembrane topology and intracellular dynamics are poorly characterized. The recent development of new, genetically-encoded probes able to detect phosphatidylserine within live cells, however, is leading to a more in-depth understanding of the biology of this phospholipid. This review aims to give an overview of the current methods for phosphatidylserine detection within cells, and some of the recent realizations derived from their use.

## 1. Introduction

**Figure 1.**
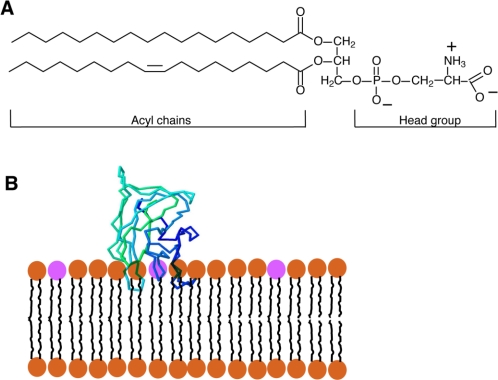
**(A)** *Diagram of the structure of a prototypical phosphatidylserine (PS),* a glycerophospholipid, with saturated and unsaturated fatty acyl chains. Note that at physiological pH the head-group bears one net negative charge. **(B)** *Representation of the interaction between the C2 domain of lactadherin (LactC2; blue-green* ®*-barrel structure) with PS in a membrane bilayer.* The head-groups of PS are shown in fuchsia, other lipids are brown. Note the three ‘fingers’ of the LactC2 structure that contain hydrophobic amino acids and are thought to reach the hydrophobic region of the membrane bilayer. The structure of LactC2 (code 3BN6) is derived from the RSCB Protein Data Bank (http://www.rscb.org/) [2].

Phosphatidylserine (PS) is a glycerophospholipid present in the membranes of all eukaryotic cells. Like the majority of glycerophospholipids, PS has a glycerol backbone esterified on the *sn*-1 and *sn*-2 carbons of the glycerol moiety with 2 fatty acyl chains of variable length and saturation, and a phosphate group on *sn*-3. The distinguishing feature of PS is the attachment of a serine to the phosphate; the resulting combined head-group gives PS a net negative charge [Figure 1(A)]. In mammalian cells, PS is produced by the exchange of the head-group of phosphatidylcholine (PC) or phosphatidylethanolamine (PE) for serine by the enzymes PS-synthase 1 (PSS1) and PSS2, respectively. Both enzymes are found in the mitochondrial-associated membranes (MAM), specialized regions of the endoplasmic reticulum (ER) that are tightly apposed to and co-isolate with mitochondria [1]. From the MAM, PS travels to other membranes within the cell.

While PS is present in all cells, it is a comparatively minor constituent of their membranes, comprising 3–10% of the total lipids [1]. However, this low relative abundance of PS belies its importance within the cell. The best-studied roles of PS involve signalling, not within the intracellular environment, but in an extracellular context such as during apoptosis [3] and during blood clotting. Like most lipids, PS is not evenly distributed throughout all cellular membranes, nor is it always equally distributed between leaflets of a membrane bilayer [4]. In healthy cells plasmalemmal PS is exclusively on the inner (cytoplasmic-facing) leaflet due to the action of ATP-dependent aminophospholipid flippases [5]. When cells undergo apoptosis (regulated cell death) PS appears on the outside-facing (extracellular) leaflet, signalling phagocytic cells to engulf the dying cell. PS is also exposed exofacially in activated blood platelets, which prompts the binding and activation of a number of clotting factors, including factors V, VIII, X and prothrombin [6].

There is little doubt that, in addition to these extracellular functions, PS plays important roles within the intracellular environment. Indeed, a number of important intracellular proteins require PS for their proper localization and/or activation. Such proteins include the E3 ubiquitin-protein ligase NEDD4, a number of protein kinase C isoforms, several phospholipase C and D isoforms, PTEN ,an important phosphatidylinositol (3,4,5)-*tris*phosphate phosphatase, dysferlin, a protein important in muscle repair, as well as a number of synaptotagmin isoforms that are important for vesicular trafficking and fusion [7]. Additionally, it is known on the whole that PS is important, as mice with a complete loss of ability to synthesize PS are not viable [8], and though yeast are able to survive without PS synthesis, their growth is greatly impaired [9]. However, despite the obvious importance of intracellular PS, its distribution, dynamics and function have not been thoroughly investigated. This is attributable, at least in part, to methodological limitations.

Early attempts to study PS relied upon cellular disruption and organellar purification, followed by chemical determination of the total PS content. Clearly, more subtle and sensitive techniques are required to fully understand the biology of PS. The ability to detect PS in intact cells and tissues, and to monitor its dynamics by non-invasive means is essential to decipher the physiology of PS. This review will discuss various methods that have been used to detect PS localization, highlighting a recent addition to the PS-sensing arsenal that allows for the detection of intracellular PS in live cells, and some novel findings derived from its use.

## 2. Methods Used to Date for Phosphatidylserine Detection and Characterization

### 2.1. PS-Binding Probes

One of the first methods used to detect the presence of PS involved the chemical 2,4,6-trinitrobenzenesulfonate (TNBS), which covalently reacts with amines; in biological membranes it therefore preferentially labels the aminophospholipids, phosphatidylethanolamine (PE) and PS. Because TNBS is membrane-impermeant and since the labelled lipids can be distinguished from the non-labelled PE and PS by thin layer chromatography (TLC) or mass spectrometry, TNBS has been used to assess the transmembrane topology of the aminophospholipids. Indeed, this is the approach used to establish that PS is present almost exclusively in the cytoplasmic leaflet of the plasma membrane of most mammalian cells [10,11]. TNBS has also been used to determine the sidedness of PS in intracellular organelles, such as mitochondria and the sarcoplasmic reticulum [12,13]. Despite its selectivity for aminophospholipids, TNBS is of limited use in live cells. It fails to react with PS in unperturbed, healthy cells and, even when PS is exposed during stimulation or apoptosis, long incubation times (15–60 minutes) are required for the reaction to become manifest, preventing meaningful dynamic studies on a timescale faster than hourly. Even in the case of fractionated cells, the probe is far from perfect: homogenization procedures and separation of organelles prior to labelling can potentially alter lipid sidedness and may breach the permeability barrier on which the sidedness determinations rely [14].

The use of antibodies to specifically recognize PS has also been considered. Such antibodies have been used successfully to detect PS on the outer leaflet of the membrane of apoptotic cells or activated platelets [15]. However, while anti-PS antibodies can be specific, there is also a significant risk of cross-reactivity with other phospholipids [16], so batch-specific characterization must be undertaken. More importantly, to immunostain PS in organellar membranes the cells must be fixed and permeabilized, in order to grant the antibodies access to the cell interior. Because permeabilization involves treatment with detergents or organic solvents that disrupt lipid architecture, it is not suitable for reliable PS detection. Additionally, the need for fixation precludes dynamic PS studies. While microinjection can in principle be used to deliver antibodies into live cells, the inability to wash the excess (unbound) antibodies prevents definition of the fraction bound to PS.

Currently, the most commonly used probe for PS detection is annexin A5, a member of the annexin family of Ca^2+^-dependent, non-covalent lipid-binding proteins. Annexin A5 binds all negatively charged lipids with relatively high affinity; however as PS is by far the most abundant negatively charged lipid (particularly on the external surface of apoptotic cells), annexin binding is generally associated with PS. Annexin A5 is readily available for purchase as a fluorescently-tagged protein, and is used extensively for detection of exofacial PS by flow cytometry or microscopy.

As in the case of antibodies, delivery of exogenous annexin to the cell interior is limited by the need for fixation and permeabilization. However, because the sequence of annexin is known, it is in principle possible to generate an annexin A5-GFP (green fluorescent protein) chimeric construct [17] for endogenous expression in transfected cells. Unfortunately, annexin is capable of binding PS only when free calcium is comparatively high: concentrations >10^−5^ M are required for proper recognition of PS [18]. Such concentrations are at least two orders of magnitude higher than the prevailing cytosolic [Ca^2+^] [19]. While it is possible to manipulate intracellular [Ca^2+^] by means such as the application of ionophores, the physiological consequences of such manipulations are deleterious and can alter the native PS distribution. Indeed, the concentrations of [Ca^2+^] required to facilitate annexin binding are known to stimulate scramblases that disrupt lipid asymmetry [20]. These considerations rule out the use of annexin A5 as a reliable probe for detection of internal PS in intact live cells.

### 2.2. Fluorescent Analogues

Another method commonly used to examine PS within the cellular environment is the use of fluorescently-labelled PS analogues. 7-nitro-2-1,3-benzoxadiazol-4-yl (NBD) is an aromatic fluorescent compound that, when attached to the end of a shortened (usually 6 or 12 carbon) *sn*-2 acyl chain, can be used to monitor the distribution and dynamics of PS without altering the unique structure of the PS head group, or having to contend with the promiscuous binding of the probes mentioned above. NDB-PS analogues are also easy to use, as they are readily loaded into cellular membranes, either by liposome transfer or using albumin as a carrier.

NBD-PS has been useful in determining specific features of the physiology of PS, such as the process whereby it is translocated (flipped) across the plasma membrane, and has aided in the identification of the responsible proteins [21–23]. On the other hand, there are a number of undesirable features that preclude the use of NBD-PS for long-term cellular imaging and thorough characterization of PS dynamics. Firstly, NBD photobleaches extremely rapidly, thus severely limiting the imaging period [24]. Additionally, the strong dipole moment of the NBD moiety forces the acyl chain to which it is attached to loop back towards the aqueous interphase  [25–27]. The resulting distortion of the labelled PS-like molecule alters the packing of the endogenous annular lipids, modifying the behaviour of the bilayer. This likely explains why NBD-PS is readily removed from membranes by albumin, while native PS and other natural lipids are refractory to extraction. How well NBD-PS mimics the behaviour of endogenous PS is therefore contentious. Its altered behaviour may explain why studies of intracellular trafficking using NBD-PS have been inconclusive, suggesting the existence of cellular transport-independent processes [22].

Another fluorophore, BODIPY, can also be used to label lipids without disruption of the chemical nature of the head group. The greater photostability and hydrophobicity of BODIPY hold promise for the study of PS though, to our knowledge, the use of acyl-labelled BODIPY derivatives of PS has not been reported to date.

## 3. Fluorescent Lactadherin C2—A New Genetically Encoded Sensor

Recently, a new PS-sensitive probe was developed that circumvents a number of the hurdles presented by the probes discussed above. The new probe is based on the glycoprotein lactadherin, a PS-binding protein which has several functions that include surrounding and stabilizing the phospholipid bilayer that coats triglyceride globules in breast milk, supporting the fusion of sperm with oocytes, and assisting in the identification of apoptotic cells for engulfment by activated macrophages [28]. Initial studies showed that lactadherin is capable of binding anionic lipids [29] through its C2 domain, which is structurally related to the discoidin-type C2 domains of coagulation Factors V and VIII. It is now clear that discoidin-type C2 domains selectively recognize PS and, importantly, they bind the phospholipid head group in a calcium-independent manner [30]. Despite the similar name, discoidin C2 domains—like that of lactadherin—are not related to the protein kinase C-type C2 domains, which bind PS and often also other anionic lipids in a calcium-dependent manner [31]. The crystal structure reveals that discoidin C2 domains like that of lactadherin contain projecting loops with hydrophobic side chains (“hydrophobic spikes”) that insert into the hydrophobic domain of the lipid bilayer, while hydrophilic residues located more deeply interact with the head group of PS [Figure 1(B)] [2,32]. While the stereospecificity for PS is conferred by the hydrophilic residues near the core of the protein, the hydrophobic spikes of the C2 domain are nevertheless very important for the binding of the C2 domain; when they are mutated to hydrophilic residues, the C2 domain is no longer able to attach to the bilayer and is therefore unable to recognize PS [2].

**Figure 2.**
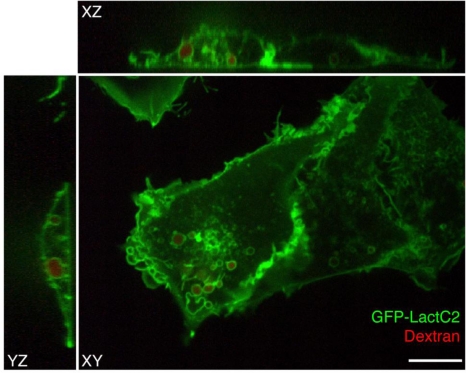
*Distribution of GFP-LactC2, a PS-specific probe, in mammalian cells.* COS-7 cells expressing GFP-LactC2 were pulsed with rhodamine-conjugated dextran followed by a 15 minute chase and were imaged live with confocal microscopy. The GFP-LactC2 probe (green) labels the plasma membrane as well as a number of internal membranes, including those positive for the fluid-phase endocytic marker, dextran (red). Bar = 10 μm.

To visualize PS in live cells, a fluorescent probe was constructed that combines the C2 domain of lactadherin with green fluorescent protein (GFP). The resulting chimera, named GFP-LactC2, is highly specific for PS and fails to bind significantly to any other lipids; it has stereospecificity for the biologically relevant isomer phosphatidyl-L-serine over phosphatidyl-D-serine, and it is quite sensitive, binding to PS more avidly than does annexin V [33–35]. Plasmids with cDNA encoding GFP-LactC2 can be readily transfected into both yeast and mammalian cells and the resulting genetically-encoded probe senses PS effectively on the cytoplasmic surface of cellular membranes, despite the prevailing submicromolar calcium concentration (Figure 2). Thus, unlike annexin V that requires artificial calcium elevation, GFP-LactC2 can be used under physiological conditions. This has enabled the first reliable, long-term measurements of PS distribution and dynamics in intact cells.

It is noteworthy that, to date, GFP-LactC2 has only been expressed within the cytosol of the cells. Because the probe is relatively large and membrane-impermeant, it can therefore only label the cytoplasmic surfaces of the cellular membranes and conveys no information regarding the presence or absence of PS in the inner (luminal) aspect of endomembrane organelles or on the outer surface of the plasma membrane. This limitation could be overcome, in principle, by adding intraorganellar-targeting domains to the sequence of the genetically-encoded probe. In these cases however, the user must ascertain that luminal free vs. bound probe can be distinguished, not an easy task in small/flat organelles such as the Golgi complex or endosomes. Table 1 highlights the advantages and disadvantages of GFP-LactC2, as well as the other methods of PS detection discussed above.

## 4. Recent Findings

The GFP-LactC2 probe was introduced very recently and its use has been reported in only a handful of studies. The initial study, which formally introduced the probe and validated its specificity, showed that, as expected, GFP-LactC2 was recruited to the plasma membrane. Indeed, the surface membrane is thought to be the largest cellular repository of PS. However, intense labelling of endosomal membranes was also noted. Early and late endosomes, as well as lysosomes were distinctly labelled [33]. Using probes that detect the surface charge, this report also showed that PS is at least partially responsible for conferring net electronegativity to these organelles. The negative surface charge contributed by PS is most likely an important factor in the recruitment of amphiphilic proteins with polycationic domains, such as K-Ras, Rac1 and c-Src [33].

GFP-LactC2 was also used to demonstrate that the limiting membrane of phagosomes is similarly rich in PS. Phagosomes are vacuoles formed when cells of the innate immune system engulf invading microorganisms, apoptotic cells or other particulate foreign bodies. As in the case of the plasma membrane and endocytic organelles, the presence of PS was associated with net electronegativity, and the surface charge was proposed to account for the recruitment of cationic protein effectors [16]. Importantly, this study found that the PS content of phagosomes varies depending on the nature of the invading microbe. Pathogenic bacteria such as *Legionella pneumophila* and *Chlamydia trachomatis* infect macrophages and survive inside them by co-opting the machinery that the host cells routinely use to eliminate other bacterial species. To avert killing, *Legionella* and *Chlamydia* alter the maturation program that converts the phagosome into an effective microbicidal organelle. Use of the GFP-LactC2 probe revealed that pathogen-induced re-routing of the maturation pathway entails depletion of PS, with the attendant loss of polycationic protein effectors.

In a recent study GFP-LactC2 was used to analyze the fate of another intracellular pathogen, *Salmonella enterica* serovar Typhimurium [36]. In this case a specific bacterial effector protein, SopB, was shown to induce a change in the net charge of the outer leaflet of the membrane of the *Salmonella*-containing vacuole. Along with changes in phosphoinositides, alterations in PS content were associated with a loss of charge that in turn affected the fate of the invasion vacuole. Finally, PS seems to play a crucial role also in the interaction between *Helicobacter pylori* and the cells of the infected host. The pathogen effector and oncogene CagA that is secreted by *Helicobacter pylori* directly binds PS. This specific interaction is required for proper localization of CagA to the inner leaflet of the plasma membrane, where it effects activation of the tyrosine phosphatase SHP-2 [37]. By a process that is less clearly understood, PS also appears to be involved in the entry of *H. pylori* into epithelial cells.

The mechanism(s) whereby PS can influence membrane function are not yet well defined. However, the importance of this unique anionic phospholipid is highlighted by recent preliminary observations in a yeast strain that lacks PS-synthase, the enzyme responsible for PS synthesis. In these mutants, which lack detectable PS, vacuolar acidification is impaired [16]. Because acidification is regarded as a key event in membrane traffic [38], alterations in pH associated with PS could have important ramifications in membrane targeting, fusion and fission.

## 5. Conclusions and Outlook

While the importance of PS has been recognized or at least suspected for a long time, studies into the physiology of this lipid have been hampered by technical limitations. The inability, until recently, to detect and visualize native PS inside live cells precluded the analysis of its distribution and dynamics. The realization that discoidin-type C2 domains can selectively detect PS, together with the elucidation of their sequence, has now enabled us to design and implement genetically-encoded fluorescent probes to detect PS in live cells. In our hands, GFP-LactC2 provides a new and powerful tool to visualize PS in organellar membranes, as well as at the plasma membrane.

The use of discoidin C2 and related probes is, however, in its infancy and currently has limitations. To date, soluble GFP-LactC2 expressed in the cytosol has been used to detect PS. Using this paradigm only the PS exposed on the cytoplasmic surface of cellular membranes is probed; no information can be gleaned about any PS potentially present in the inner (luminal) monolayers of organelles or on the outer surface of the plasmalemma. The possibility exists, however, to deliver LactC2 or similar probes to the lumen of defined organelles by attachment of specific targeting sequences such as those that direct proteins to the interior of mitochondria or peroxisomes. We are currently experimenting with a variant of GFP-LactC2 modified to include a secretion signal (the pre-pro peptide signal from insulin) and a retention signal (KDEL) to target the probe to the lumen of the endoplasmic reticulum. We foresee difficulties in differentiating bound from free probe, considering that the lumen of the reticulum network is below the optical resolution of light microscopy [39]. However, biophysical techniques such as FRAP (fluorescence recovery after photobleaching) and FCS (fluorescence correlation spectroscopy) may prove more informative, since the mobility of GFP-LactC2 should be markedly affected when the probe is bound to PS.

The probes could in principle be used also for ultrastructural analysis of PS by electron microscopy. This could entail either conventional immunogold labelling of sections of cells expressing GFP-LactC2 endogenously. Alternatively, thin sections of frozen samples could be overlaid with recombinant LactC2, in which case both the cytosolic and luminal aspects of the membranes would be exposed to the probe. A similar approach has been used Parton and colleagues to detect phosphatidylinositol 3-phosphate, yielding valuable information on the presence of this lipid in luminal structures of multivesicular bodies [40].

Like all probes, the discoidin C2 domains sequester the substrate that they bind to, and can thereby potentially alter the physiology of the cells. The K_d_ of the binding reaction between LactC2 and PS is approximately 2–3 nM [30,35], which is only slightly lower than the K_d_ of other lipid-binding probes such as the PH domains that bind phosphatidyl inositides [41,42]. This implies that, at the concentrations prevailing in cells much of the probe would be in the bound form. While this favours sensitive detection of PS, it could in principle disturb cellular homeostasis by scavenging a physiologically necessary ligand. What is important to consider, however, is the maximal fraction of the cellular PS that could be engaged by the probe under the experimental condition used. Determinations of the total cellular content of PS, together with estimates of the cell volume, suggest that the cellular concentration of PS approximates 300 μM  [43,44]. If, as is generally the case, the probe is expressed at submicromolar concentrations, then the fraction of PS scavenged should be negligible and the associated functional consequences would be minimal. Nevertheless, it is important to bear in mind the mol ratio of the probe and its intended target.

In conclusion, the pre-existing technical barrier to advance our knowledge of the biology of PS has been lowered, though not yet eliminated, clearing the way for more rapid progress in the understanding of this important lipid species.

## Figures and Tables

**Table 1.** Advantages and Disadvantages of PS detection methods.

**PS Detection Method**	**Advantages**	**Disadvantages**
Biochemical fractionation followed by mass spectrometric identification	Multiple organelle detection; Can distinguish various PS species	Large amounts of material required; No sidedness information; Liable to contamination Only useful for external PS detection; Limited selectivity; Slow reaction time For detection of external PS only (detection of internal PS requires fixation and permeabilization) Imperfect mimic of endogenous PS; Liable to metabolic conversion; Rapid photobleaching Only external PS readily measurable (internal labelling requires supra-physiological calcium levels). Requires transfection or microinjection; May scavenge endogenous PS
PS-reactive compounds (e.g., TNBS)	Yields sidedness information	
Antibodies	High selectivity	
NBD-PS	Yields dynamic information; Useful in intact cells	
Annexin V	Sensitive; Available with multiple tags for easy detection; Dynamic information obtainable	
Discoidin-C2 (GFP-LactC2)	Highly specific; Provides dynamic information; Useful for intracellular imaging.	
